# Effects of Aerobic Exercise on Anxiety Symptoms and Cortical Activity in Patients with Panic Disorder: A Pilot Study

**DOI:** 10.2174/1745017901814010011

**Published:** 2018-02-21

**Authors:** Eduardo Lattari, Henning Budde, Flávia Paes, Geraldo Albuquerque Maranhão Neto, José Carlos Appolinario, Antônio Egídio Nardi, Eric Murillo-Rodriguez, Sérgio Machado

**Affiliations:** 1 Laboratory of Panic and Respiration, Institute of Psychiatry, Federal University of Rio de Janeiro (IPUB/UFRJ), Rio de Janeiro, Brazil; 2 Faculty of Human Sciences, Medical School Hamburg, Kaiserkai 1, 20457 Hamburg, Germany, Lithuanian Sports University, Kaunas, Lithuania, Physical Activity, Physical Education, Health and Sport Research Centre (PAPESH), Sports Science Department, School of Science and Engineering, Reykjavik University, Reykjavik, Iceland; 3 Physical Activity Sciences Post-Graduate Program (PGCAF), Salgado de Oliveira University, Niteroi, Brazil; 4 Federal University of Rio de Janeiro (IPUB/UFRJ), Rio de Janeiro, Brazil; 5 Laboratorio de Neurociencias Moleculares e Integrativas. Escuela de Medicina, Division Ciencias de la Salud, Universidad Anahuac Mayab. Merida, Yucatan. Mexico; 6 Laboratory of Physical Activity Neuroscience, Physical Activity Sciences Post-Graduate Program (PGCAF), Salgado de Oliveira University, Niteroi, Brazil

**Keywords:** Exercise, Aerobic exercise, Anxiety, EEG frontal asymmetry, Anxiety Disorders, Panic Disorder

## Abstract

**Background:**

The effects of the aerobic exercise on anxiety symptoms in patients with Panic Disorder (PD) remain unclear. Thus, the investigation of possible changes in EEG frontal asymmetry could contribute to understand the relationship among exercise, brain and anxiety.

**Objective:**

To investigate the acute effects of aerobic exercise on the symptoms of anxiety and the chronic effects of aerobic exercise on severity and symptoms related to PD, besides the changes in EEG frontal asymmetry.

**Methods:**

Ten PD patients were divided into two groups, Exercise Group (EG; n=5) and Control Group (CG; n=5), in a randomized allocation. At baseline and post-intervention, they submitted the psychological evaluation through Panic Disorder Severity Scale (PDSS), Beck Anxiety Inventory (BAI), Beck Depression Inventory-II (BDI-II), EEG frontal asymmetry, and maximal oxygen consumption (VO_2_max). On the second visit, the patients of EG being submitted to the aerobic exercise (treadmill, 25 minutes, and 50-55% of heart rate reserve) and the CG remained seated for the same period of time. Both groups submitted a psychological evaluation with Subjective Units of Distress Scale (SUDS) at baseline, immediately after (Post-0), and after 10 minutes of the rest pause (Post-10). The patients performed 12 sessions of aerobic exercise with 48-72 hours of interval between sessions.

**Results:**

In EG, SUDS increased immediately after exercise practice and showed chronic decrease in BAI and BDI-II as well as increased in VO_2_max (Post-intervention).

**Conclusion:**

Aerobic exercise can promote increase in anxiety acutely and regular aerobic exercise promotes reduction in anxiety levels.

## 1 INTRODUCTION

Panic Disorder (PD) can be characterized by recurrent and unexpected Panic Attacks (PAs) where at least one of the attacks was followed by a persistent concern about new attacks and their consequences for a period of one month or more [1]. Lifetime prevalence of PD is 1.7% with a median age of onset by 32 years and 80.4% of persons with lifetime PD had a comorbid mental disorder [2]. Besides PD associations with mental disorders, it is also associated with clinical comorbidities [3], loss of productivity, well-being, social contact and self-realization [4], causing considerable health costs [5].

First-line treatments for PD are selective serotonin reuptake inhibitors, serotonin-noradrenaline reuptake inhibitors, calcium channel modulator pregabalin, tricyclic antidepressants [6], and benzodiazepines [7]. However, long-term tolerability issues associated with antidepressants and benzodiazepines exposure should also be carefully considered [8]. In addition, 20% of PD patients are refractory to standard treatments [9]. Within this context, potential treatment options for PD patients, such as Cognitive Behavioural Therapy (CBT) and other variants of behavior therapy [6], as well physical exercise [10] have attracted attention in the literature.

With respect to physical exercise, more specifically aerobic exercise, studies has been used it in PD patients demonstrating acute anxiolytic and antipanic effects [11, 12]. Esquivel *et al.* [11] compared two conditions of aerobic exercise, high intensity versus low intensity, on the antipanic effects after inhaling 35% of carbon dioxide (CO_2_). PA reactions to CO_2_ were lower in patients who underwent high intensity aerobic exercise compared to low intensity. Results also demonstrated the capacity of aerobic exercise to minimize anxiety symptoms in PD patients after use of substances that induce a PAs, such as caffeine [13], cholecystokinin-tetrapeptide (CCK-4) [14], and CO_2_ [11]. However, other studies showed that aerobic exercise induces an increase in anxiety in PD patients [14, 15]. Rief and Hermanutz [15] found that aerobic exercise, during two minutes and intensity of 75 watts, was sufficient to lead to increase in anxiety in PD patients. Strohle *et al.* [14] also demonstrated that 30 minutes of aerobic exercise, with an intensity of 70% of maximal oxygen consumption (VO_2_max), generated increase in anxiety. This alteration was observed in somatic symptoms [14], suggesting that patients with PD are more vulnerable to experiencing somatic symptoms after aerobic exercise [15]. However, Martinsen *et al.* [16] showed that twenty-four hospitalized PD patients completed supramaximal exercise tests (3-minute steady-state exercise test and workload at 110%) and all experienced high values of lactate (10.7 mmol/L), but only 1 patient experienced a PA during the exercise testing. Thus, the acute effect generated by aerobic exercise on anxiety symptoms in PD patients remains unclear.

Another question persists if aerobic exercise promotes, in the long term, anxiolytic effect in PD patients [17]. Meyer *et al.* [17] submitted patients to three experimental groups, clomipramine, aerobic exercise or placebo. They showed that treatment with clomipramine was more effective in disease severity and anxiety symptoms compared to aerobic exercise. Aerobic exercise was more effective in anxiety symptoms compared to placebo only at the end of the study (*i.e.*, week 10). Bandelow *et al.* [18] evaluated three treatment modalities: running, clomipramine or placebo. Treatment efficacy was measured with the Panic and Agoraphobia Scale (P &A) and other rating scales. According to the P &A and other scales, both exercise and clomipramine lead to significant decrease in symptoms compared to placebo treatment. Again clomipramine was significantly more effective and improved anxiety symptoms significantly earlier than exercise. In a study carried by Wedekind *et al.* [19] patients with PD were submitted to four experimental groups, aerobic exercise with paroxetine, relaxation with paroxetine, aerobic exercise with placebo, or relaxation with placebo. The results showed response and remission rates only in paroxetine group. No improvement was observed in aerobic exercise. Thus, the ability of aerobic exercise to promote long-term improvements in anxiety in PD patients is questionable.

Of particular interest to the present study are models that link frontal brain asymmetry to individual differences in affective style. This frontal asymmetry particularly postulated within the alpha frequency band (8-12 Hz) is the main base of Davidson model [20]. The model proposed by Davidson *et al.* demonstrates that left frontal areas of the brain mediating the experience of positive emotions and approach behaviors, and right frontal areas of the brain mediating the experience of negative emotions and withdrawal behaviors were associated with higher activation of the right frontal cortex and positive affective responses were associated with higher activation of the left frontal cortex [20]. These patterns of frontal Electroencephalographic (EEG) asymmetry may serve as an index of risk for a variety of emotion-related disorders. Patients with major depression [21] and social anxiety [22, 23] have been shown significant relative elevations in right frontal brain activity when assessed during resting states or periods of acute emotional provocation, supporting these theoretical predictions [24].

According to our knowledge, there are no studies published that investigated changes in EEG frontal activity among individuals with PD before and after aerobic exercise. In health subjects, Petruzzelo and Tate showed that EEG frontal asymmetry was predictive of positive affect immediately after exercise using intensities close to the ventilatory threshold (70% of VO_2_max) [25]. However, EEG frontal asymmetry measured followed by aerobic exercise conditions, was influenced by three exercise intensities (45%, 60% and 75% VO_2_max), and associated with increase in the vigor imposed no differences between three intensities [26]. Although no studies to date have investigated patterns of change in EEG frontal activity among individuals with PD before and after aerobic exercise, Lattari *et al.* [27] demonstrated that aerobic exercise did not change the frontal asymmetry in health subjects.

Thus, the acute and chronic effects generated by aerobic exercise on anxiety symptoms in patients with PD remain inconclusive. In addition, the intensity of effort that promotes anxiolytic effects remains unanswered. Acutely, the intensity of aerobic exercise has been reported objectively [11, 14, 15], but it is not known until now whether it causes an increase or decrease in anxiety in patients with PD. Chronically, research used this variable in an arbitrary form [19, 28], only one study that determined the intensity of effort in an objective form [10]. Details on the intensity, frequency and duration may further support the clinical administration of the exercise in patients with panic disorder [29]. Of particular interest to the present study is the investigation patterns of change in EEG frontal activity among individuals with PD before and after aerobic exercise and to evaluate predictions on levels of anxiety.

Besides, the study had the following objectives: 1) to investigate the acute effects of aerobic exercise on symptoms of anxiety; 2) to investigate the chronic effects of aerobic exercise on severity and symptoms related to PD, besides the changes on EEG frontal asymmetry. Our hypothesis for acute effects is that exercise compared to resting condition will provide further increases in anxiety immediately after, with significant reductions after 10 minutes of recovery. The regular practice of aerobic exercise will have greater reductions in severity and symptoms related to PD, as well as a increase in frontal asymmetry compared to control group.

## 2 METHODS

### 2.1 Patients

The recruited patients had a diagnosis of PD according to Diagnostic and Statistical Manual of Mental Disorders (DSM-IV-TR) (*i.e.*, unexpected PAs in the last month) [1] with at least 5 years of disease duration. The patients should be symptomatic by Panic Disorder Severity Scale (“PDSS”) (> 10 points, moderately ill). Diagnoses were made by an experienced psychologist using the Structured Clinical Interview for DSM-IV-TR. The exclusion criteria were: pregnancy, lactation, severe medical illness, organic brain damage, bipolar affective disorder, severe major depression, psychotic symptoms, alcohol or drug abuse, anorexia or bulimia nervosa, and regular aerobic exercise. The patients were sedentary and for participation in aerobic exercise, patients were requested to be authorizated by their cardiologist. Patients were not allowed to undergo additional psychological treatment during the study. Patients should be in continued treatment with any psychotropic drugs at least two weeks before baseline. The patients were in regular use with the selective serotonin reuptake inhibitors 150mg/day (fluoxetine, paroxetine, and citalopram) and/or benzodiazepines 3mg/day (clonazepam). Ten patients were eligible for the study in accordance with the inclusion and exclusion criteria. Each patient was clarified of all the experimental procedures and signed a written consent form, with the experiment was approved by the institutional ethics committee of the Federal University of Rio de Janeiro. The patients characterization can be observed in Table **1**.

### 2.2 Experimental Procedures

On the first visit (1^st^ day) to the Panic and Respiration of Laboratory, the patients were submitted to a battery of psychological evaluation (diagnosis of PD with DSM-IV-TR, Panic Disorder Severity Scale, Beck Anxiety Inventory, and Beck Depression Inventory II), recording the electroencephalographic activity (resting EEG asymmetry), and determining the maximum oxygen consumption (VO_2_max) (Baseline). Ten patients were included and randomly assigned to two groups, Exercise group (n=5) and Control group (n=5).

On the second visit (2^nd^) to the laboratory, the patients of exercise group were submitted to the aerobic exercise protocol (see “Aerobic Exercise Protocol”). Patients in the control group remained seated comfortably in an armchair for the same period of time. Both groups were submitted a psychological evaluation with Subjective Units of Distress Scale (SUDS) at baseline, immediately after (Post-0), and after 10 minutes of resting (Post-10). These experimental procedures were performed to verify the acute effects of aerobic exercise on anxiety symptoms.

During the chronic experimental phase, patients performed 12 sessions of aerobic exercise (2^nd^ to 13^th^ day, see in the “Aerobic Exercise Protocol”). Aerobic exercise was performed with 48-72 hour rest interval between sessions. At the end of the chronic experimental phase (14^th^ day), the same evaluations of the first visit (1^st^ day) were performed, except for DSM-IV-TR (Post-intervention). Psychological evaluations were made by a senior psychologist. Electroencephalographic recording and VO_2_max assessment was made by an experienced professional of physical education. The aerobic exercise sessions were also accompanied by the same physical education professional. Patients in the control group were instructed to not exercise during the chronic experimental phase and remain on medication. The experimental procedures are shown in Fig. (**1**).

### 2.3 Aerobic Exercise Protocol

All patients performed the training routine without interruptions and in the same calibrated treadmill (INBRAMED, Brazil). The patients performed 12 sessions with 48-72 hours of the interval between each one. They received information about the training procedures and also were encouraged to work until the end of the set time, but advised that they could also abort the training session to signal complications or fatigue. The intensity settings occurred at 45-50% of HRR for warm up, 50-55% of HRR for training and 45-50% of HRR for cool down. The warm up and cool down were performed with two minutes and thirty seconds, respectively, and aerobic training was performed on twenty minutes, totalizing 25 minutes. This prescription of aerobic exercise is in agreement with the American College of Sports Medicine [30].

### 2.4 Panic Disorder Severity Scale (PDSS)

The Panic Disorder Severity Scale (PDSS) is a questionnaire developed for measuring the severity of panic disorder [31].The PDSS consists of seven items that assess: 1) Panic frequency; 2) Distress during panic; 3) Panic-focused anticipatory anxiety; 4) Phobic avoidance of situations; 5) Phobic avoidance of physical sensations; 6) Impairment in work functioning; 7) Impairment in social functioning.

Each item is rated on a 5-point scale, which ranges from 0 to 4. The total scores range from 0 to 28.

### 2.5 Subjective Units of Distress Scale (SUDS)

Subjective Units of Distress Scale (SUDS) is a scale of 0 to 10 for measuring the subjective intensity of disturbance or distress currently experienced by an individual. The anxiety level has been measured with the Subjective Units of Distress Scale (SUDS) in patients with panic disorder [32-34].

### 2.6 Beck Anxiety Inventory (BAI)

Beck Anxiety Inventory (BAI) is a 21-question multiple-choice self-report inventory that is used for measuring the severity of anxiety [35]. Each answer is scored on a scale value of 0 (not at all) to 3 (severely). The BAI has been used as secondary outcomes of the anxiety in patients with panic disorder after aerobic exercise program [10, 28].

### 2.7 Beck Depression Inventory-II (BDI-II)

Beck Depression Inventory-II (BDI-II) is a 21-question multiple-choice self-report inventory that is used for measuring the severity of depressive episodes [36]. Each answer is scored on a scale value of 0 (not at all) to 3 (severely).

### 2.8 Resting EEG Asymmetry

EEG signal was recorded by NeuroSpectrum-5 (Medical Instruments, São Paulo, Brazil). Nineteen monopolar electrodes were placed in the frontal areas (Fp1, Fp2, Fz, F3, F4, F7 and F8), central (Cz, C3 and C4), temporal (T3, T4, T5 and T6), parietal (Pz, and P3 P4) and occipital (O1 and O2) according to the International System 10/20 protocol [37]. Two other electrodes (*i.e.*, A1 and A2) were positioned in the earlobes with reference function (bi-auricular). The EEG suffers 0.05 Hz analog filtering (high pass) and 500 Hz (low-pass). EEG Sampling Rate is 5000 Hz and noise level is less than 0.3 μV. We used a digital notch filter 60 Hz and high-pass filters also on 0.5 Hz and low pass at 35 Hz.

The impedance levels of each electrode were observed, which should be between 5 and 10 KOhms (KΩ). EEG analyzes were performed in Neuron-Spectrum.NET-5 software. Initially, the artifacts were automatically removed, as spikes and sharp waves. In addition, the estimation of signal components by Independent Component Analysis (ICA) was applied to minimize artifacts. A visual inspection of the data to remove artifacts was performed, such as sweating and muscle tension.

Through Fast Fourier Transform (FFT), that refers to a signal analysis that repeats itself at regular time intervals, can set how much energy (power) is in each frequency band. The spectral absolute power was used with 1-s window for time of EEG, free of artifacts (spectral resolution of 0.25 Hz). The digital EEG systems of Neuron-Spectrum-5 series allow EEG recording in standard ranges delta, theta, alpha, beta and gamma. Epochs were extracted in the alpha frequency band (8-14Hz). The log transformation was applied (natural log ln) on the power values, since EEG power have not a normal distribution between subjects. This procedure results in a near normal distribution. EEG data were analyzed for cortical asymmetry according to Davidson hypothesis [20]. The basic mathematical calculation to compute the asymmetry was expressed by the equation: Resting EEG Asymmetry = InF4- InF3; the choice of Fronto-medial electrodes counterparts (F4-F3) was based on the prevalence of this measure in the literature related to exercise and association between asymmetry with reduction in anxiety scores [22, 23].

### 2.9 Determination of the Maximal Oxygen Consumption (VO_2_Max)

For determination of VO_2_max, the submaximal protocol of Oliveira *et al.* was used [38]. Thus, patients remained seated for 10 min at rest, and in acclimatized environment (20° - 21°C). After 10 minutes of rest, the resting heart rate (HRrest) was measured by a specific monitor (model RS800, Polar®, Finland). The maximal Heart Rate (HRmax) was also determined by the following equation: 220 – age (years).

The purpose of the test is to achieve the intensity corresponding to approximately 65% of Heart Rate Reserve (HRR). To determine the HRR, the equation was used: (HRmax - HRrest) x intensity + HRrest. Once hit, this intensity of effort is maintained for 6 minutes, characterizing a steady-state. It is expected that in the end of this stage FC was stabilized at approximately 70% of HRR. Intensity corresponds to the intensity that the patient should have reached during the test (65% of HRR) and maintain in steady state (70% of HRR) [39]. The submaximal protocol was performed with an initial warm up of 3 minutes on a treadmill (INBRAMED, Brazil), with a metabolic increment equivalent to 1 MET per minute, provided by the manipulation in the inclination of treadmill.

The VO_2_ was obtained by the walking equation: VO_2_ = [0,1 (speed) + 1.8 (speed) (inclination / 100) + 3.5], in which the speed is given in m/min. Finally, the VO_2_max was predicted by the equation: VO_2_max = [(VO_2_ - 3.5) / %HRR + 3.5], in which VO_2_max is expressed in mL.kg^-1^.min^-1^(30).

### 2.10 Statistical Analysis

Descriptive statistics (mean and standard deviation) were calculated for age, body weight, height, SUDS, PDSS, BAI, BDI-II, VO_2_max, and resting EEG asymmetry. At baseline, independent samples t-tests were used to verify the differences between the two groups (Exercise group *vs* Control group). A 2 × 2 mixed factor analysis of variance was used to test for differences between control group and exercise group (between-group effects) and differences between baseline, immediately after (Post-0), and 10 minutes post-intervention (Post-10) (within-group effects) for SUDS. A 2 × 2 mixed factor analysis of variance was used to test for differences between control group and exercise group (between-group effects) and differences between baseline post-intervention (within-group effects) for PDSS, BAI, BDI-II, VO_2_max, and resting EEG asymmetry. Post-hoc analysis was performed using the Bonferroni to assess the effects within each group. Assumptions of the homogeneity of variance and residual normality were tested by using the Levene’s and Shapiro-Wilk tests respectively. If assumptions were not met, changes in scores for those outcome measures were computed. Depending on the normality of the data, change scores for each group were compared by using independent samples t-test or Mann-Whitney U tests. Analyses were led separately for all the outcome variables including Panic SUDS, PDSS, BAI, BDI-II, VO_2_max, and resting EEG asymmetry. The level of significance was set at p ≤ 0.05.

Effect size analysis for each group was calculated by using Cohen’s d. The following measures were used: PDSS, BAI, BDI-II, VO_2_max, and resting EEG asymmetry. Given that the calculation was for within group effects, we correlated both means and used Morris &DeShon’s [40] equation. Calculations were completed by using the G*POWER software (version 3.1). Effect sizes were classified as trivial (d <0.19), small (d = 0.20-0.49), moderate (d = 0.50-0.79), large (d = 0.80-1.29) and very large (> 1.30) [41].

## 3 RESULTS

At baseline, there were no significant group differences regarding age, height, SUDS, PDSS, BAI, BDI-II, VO_2_max, and resting EEG asymmetry (p > 0.05), with the exception of the body weight (p=0.03). Thereby, it can be assumed that participants in both groups were similar in relevant variables at the moment the intervention began (Table **1**).

### 3.1 SUDS

**Fig. (2):**
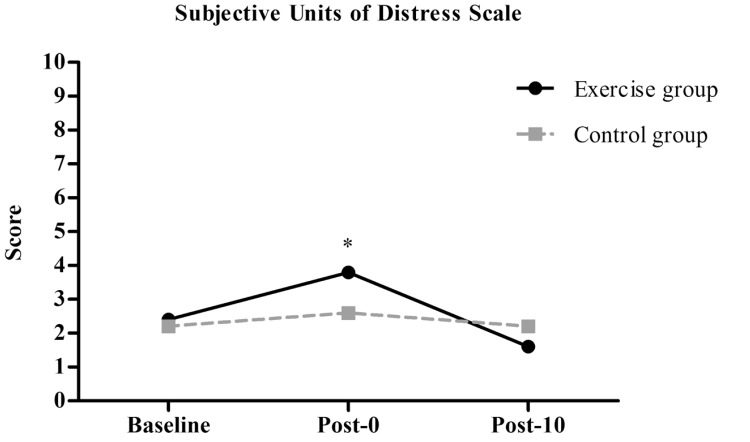
Acute effects of exercise on SUDS.

Mixed analysis of variance showed significant group by time interaction (p = 0.026), and also significant main effects for time (p=0.001) for SUDS. Post-hoc analysis revealed a significant increase in SUDS in the exercise group after the intervention (Post-0= 3.8±0.8) compared to baseline (Baseline= 2.4±1.1; p=0.04) and after 10 minutes of resting (Post-10= 1.6±0.5; p = 0.003) (Fig. **2**). There were no significant differences between the exercise and control groups at baseline (Exercise Group=2.4±1.1 *vs* Control Group= 2.2±0.8, p=0.76), immediately after (Exercise group=3.8±0.8 *vs* Control Group= 2.6±1.8, p=0.21) and after 10 minutes of resting (Exercise group=1.6±0.5 *vs* Control group= 2.2±1.0; p=0.30).

### 3.2 PDSS

The results showed no significant group by time interaction (p=0.41) and no significant main effects for time (p=0.18) for PDSS. Descriptive data are described in Table **2**.

### 3.3 BAI

**Fig. (3):**
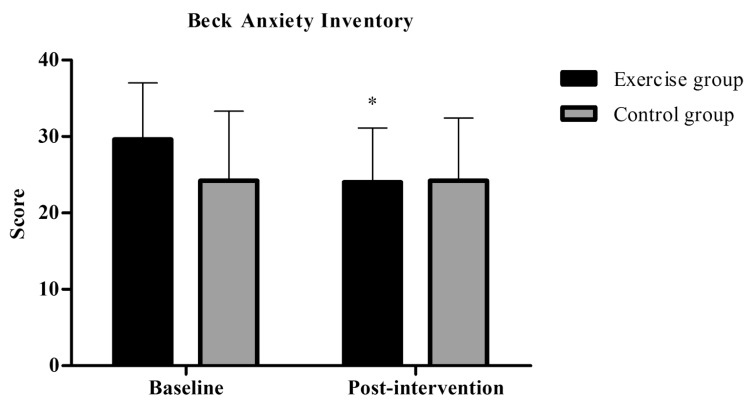
Between and within-group comparisons for BAI.

Mixed analysis of variance showed significant group by time interaction (p=0.02), and main effects for time (p=0.02) for BAI. Post-hoc analysis revealed a significantly decreased score in BAI in the exercise group post-intervention (M±SD= 29.6±7.4) compared to baseline (M±SD= 24.0±7.1, p=0.005) (Fig. **3**).

**Fig. (4):**
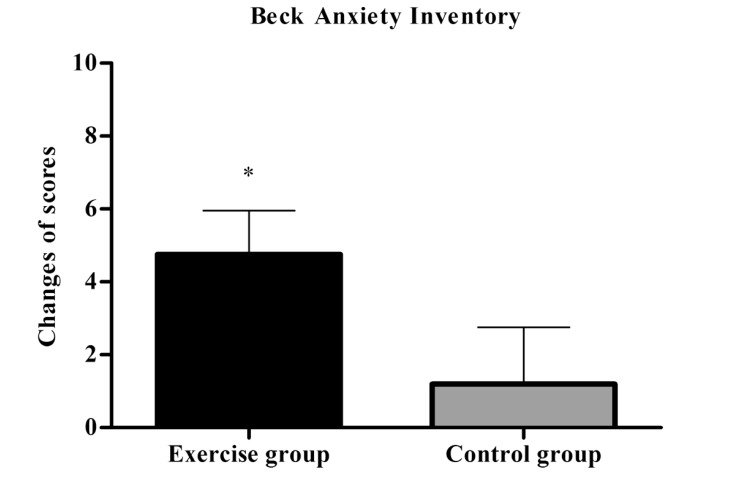
Independent samples t-test for changes in BAI scores.

Independent samples t-test indicated significant between-group differences regarding BAI (Exercise Group=5.6±3.9 *vs* Control Group= 0.1±2.3, p = 0.02), showing that the exercise group had greater reductions in the scores (Fig. **4**). Moreover, only the exercise group showed very large effect size (1.40) within group. Descriptive data can be observed in (Table **2**).

### 3.4 BDI-II

Mixed analysis of variance showed significant group by time interaction (p = 0.04), and no significant main effects for time (p = 0.09) for BDI-II. Post-hoc analysis revealed a significantly decreased score in BDI-II in the exercise group post-intervention (M±SD= 15.8±9.4) compared to baseline (M±SD= 21.6±10.7, p=0.01) Fig. (**5**). Mann-Whitney’s U test showed no between-group differences for BDI-II (Exercise group=5.8±5.3 *vs* Control group= -0.6±2.8, p= 0.09). However, only the exercise group showed large effect size (1.06) within group. Descriptive data are seen in Table **2**.

### 3.5 VO_2_max

**Fig. (6):**
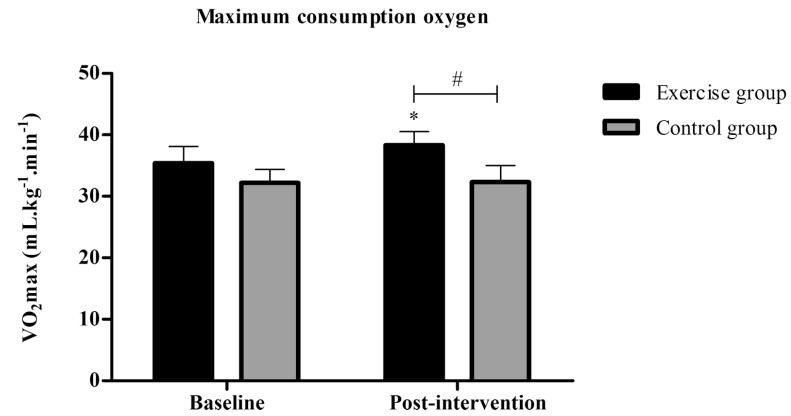
Between and within-group comparisons for VO_2_max.

Mixed analysis of variance showed significant group by time interaction (p < 0.001), and also significant main effects for time (p < 0.001) for VO_2_max. Post-hoc analysis revealed a significant increase in VO_2_max in the exercise group post-intervention (M±SD= 38.3±2.2 mL.kg^-1^.min^-1^) compared to baseline (M±SD= 35.4±2.7 mL.kg^-1^.min^-1^, p < 0.001). In addition, VO_2_max was greater in the exercise group compared to the control group in post-intervention (Exercise group= 38.3±2.2 mL.kg^-1^.min^-1^* vs *Control group= 32.3±2.7 mL.kg^-1^.min^-1^, p=0.005) (Fig. **6**).

**Fig. (7):**
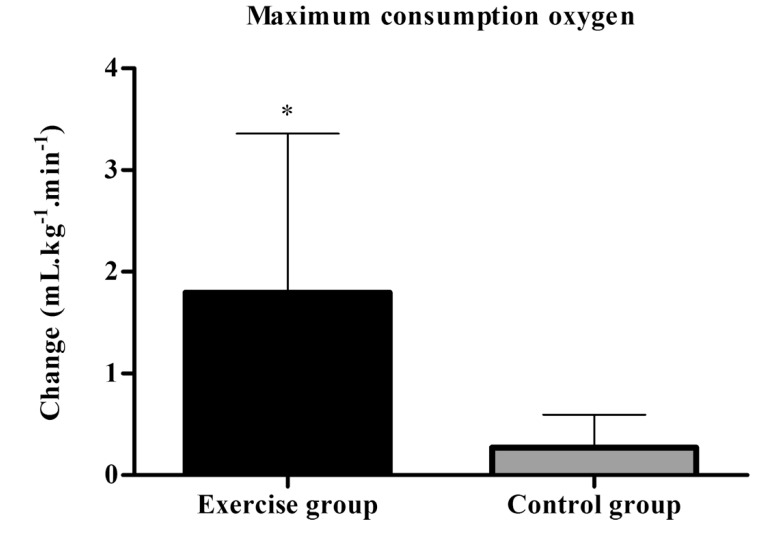
Independent samples t-test for changes in VO_2_max.

Independent samples t-test indicated significant between-group differences regarding VO_2_max (Exercise group= 2.9±0.6 mL.kg^-1^.min^-1^ *vs* Control group= 0.04±0.5 mL.kg^-1^.min^-1^, p < 0.001) (Fig. **7**). Besides, results of the effect size between (*d*= 2.43) and within groups (*d*= 4.15) showed very largely, in favor of exercise group. Descriptive data are shown in (Table **2**).

### 3.6 Resting EEG asymmetry

**Fig. (8):**
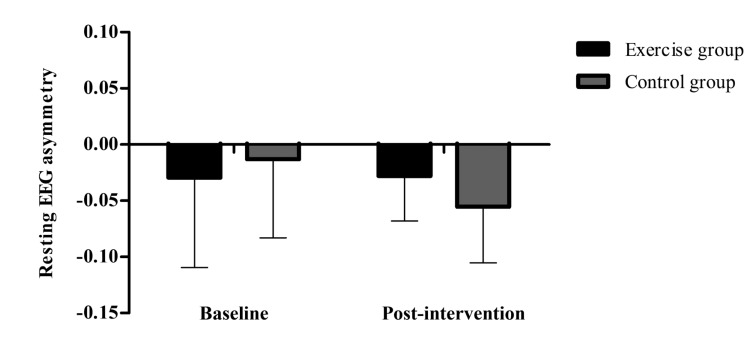
Between and within-group comparisons for resting EEG asymmetry.

Results showed no significant group by time interaction (p=0.43) and no significant main effects for time (p=0.46) for resting EEG asymmetry (Fig. **8**). Descriptive data can be seen in Table **2**.

## 4 DISCUSSION

The aim of study was to verify the acute effects of aerobic exercise on the symptoms of anxiety and to investigate the chronic effects of aerobic exercise on severity and symptoms related to PD, besides the changes caused on EEG frontal asymmetry. Acute effects of exercise provided increases in anxiety immediately after and significant reductions after 10 min of recovery. The regular practice of aerobic exercise generated greater reductions in symptoms related to PD, BAI and BDI-II. However, no changes were demonstrated in PDSS and frontal EEG asymmetry in both groups.

In fact aerobic exercise can promote increase in anxiety acutely and other researches corroborated our findings [14, 15]. Rief and Hermanutz [15] submitted patients with PD to two experimental conditions, exercise and control. The exercise was performed in the cycle ergometer, with duration of two minutes and intensity of 75 watts. The results showed that patients with PD had elevated anxiety scores after physical activation, but also after rest. Strohle *et al.* [14] also investigated the effects of quiet rest or an aerobic treadmill exercise on antipanic and anxiolytic activity in patients with PD. Aerobic exercise was performed in treadmill, 30 min at an intensity of 70% of VO_2_max. Results showed that somatic anxiety subscore were higher after exercise compared to rest and no results have been demonstrated for anxiety symptoms subscore. It is expected that some somatic symptoms increase during exercise and that responses of increase in anxiety levels are linked to this factor, as corroborated by the study of Strohle *et al.* [14]. Our findings shows this increase in anxiety may be related to possible changes in somatic symptoms. However, although aerobic exercise has promoted increased levels of anxiety, exercise exposure may provide antipanic protective effect. Strohle *et al.* [14] showed that CCK-4-induced panic attacks were less frequent after prior exercise (Exercise= 4 patients, 33.3% and Rest= 9 patients, 75%). Compared to prior rest, exercise resulted in a significantly reduced CCK-4-induced increase of the total Acute Panic Inventory (API) score and the anxiety subscore. Esquivel *et al.* [11] also demonstrated the protective effect of exercise for panic attacks and levels of anxiety. Patients participated in two experimental conditions, moderate/hard exercise (to reach and sustain on the cycloergometer 80 and 90% of their maximal heart rate for 15 minutes with or until exhaustion) or a very-light exercise (15 minutes, 1watts per kg, and 20 at 70 rpm, control group). Panic reactions to CO_2_ were smaller in patients that performed moderate/hard exercise in contrast to those that performed very-light exercise.

Due to therapeutic potential of acute aerobic exercise, this intervention non-pharmacological was investigated also chronically. Our results showed that aerobic exercise reduced anxiety after 12 training sessions and data of randomized controlled trials suggest higher sizes for the effect of exercise on anxiety [42]. Bandelow *et al.* [18] submitted the patients to three treatment conditions: clomipramine, placebo and aerobic exercise (walking or running). Both exercise and clomipramine led to a significant decrease of anxiety symptoms in according to the P&A and HAM-A, in comparison to placebo treatment. Clomipramine was significantly more effective than aerobic exercise. Similar results were found in the Broocks *et al.* [28] and Meyer *et al.* [17]. Although the anxiolytic and antipanic effects of exercise have already been empirically observed, the mechanisms of action involved remain to be elucidated. Several plausible mediators have been set forward to explain the antipanic and anxiolytic effects of exercise. Current studies have centered on mechanisms related to the brain-derived neurotrophic factor [43], atrial natriuretic peptide [12], and the serotonergic system [44].

Wedekind *et al.* [19] showed no improvement in anxiety after regular aerobic exercise. In this study, patients were divided into four experimental groups: aerobic exercise with paroxetine, relaxation with paroxetine, aerobic exercise with placebo, or relaxation with placebo. Results showed that response and remission rates were higher in the groups using paroxetine, regardless of exercise or relaxation, compared to placebo groups. Aerobic exercise showed a trend of improvement compared to the relaxation group, seen only in the fourth week. It is still necessary to establish the duration, frequency and intensity of aerobic exercise that promotes the beneficial organic adaptations to patients with PD. In our research, the aerobic exercise protocol was established according to the ACSM recommendations [30] and the control over aerobic exercise intensity has been subjective in others studies [17-19]. The intensity settings at 50-55% of HRR for training and twenty minutes of duration is recommended for sedentary subjects and low cardiorespiratory fitness [30]. Patients with PD showed lower VO_2_max, as well as lower exercise tolerance [45]. With this, our training prescription seems appropriate.

In addition to reductions in anxiety levels, our research demonstrated that regular aerobic exercise provided an increase in VO_2_max and reductions in depression scores. These results suggest that regular aerobic exercise, in comparison with control group, is associated with significant clinical improvement in PD patients. Martinsen *et al.* [46] reported the results of a protocol of 60 min of AE at 70% of VO_2_max, 3 times a week for 8 weeks, in which they observed a significant increase in VO_2_max with significant reductions in depression scores.

Our hypothesis was not confirmed for the increase in resting EGG asymmetry related to regular practice of aerobic exercise compared to control group. No changes in resting EEG frontal asymmetry were observed. To date, no study investigated changes in resting EEG asymmetry caused by regular practice of aerobic exercise. Lattari *et al.* [27] showed that acute aerobic exercise does not change resting EEG asymmetry.

A limitation of our study is the impossibility to establish double-blind conditions. Besides, our results should be interpreted with caution due to sample size. PD patients generally are afraid to do exercise because of the physiological responses that are similar to panic symptoms. However, our research was innovative in the most appropriate control of aerobic training. In addition, it was the first study to investigate the effects of aerobic exercise on resting EEG asymmetry in patients with PD.

## CONCLUSION

In conclusion, aerobic exercise can promote increase in anxiety acutely. However, regular aerobic exercise promotes reduction in anxiety levels. In addition, the regular practice of aerobic exercise promotes other interesting improvements such as maximal oxygen consumption and reduction in the symptoms of depression. The hypothesis of resting EEG asymmetry in the exercise group in post-intervention was not confirmed. Future researches should further examine how the frontal asymmetry is associated with changes in anxiety after the regular practice of aerobic exercise.

## Figures and Tables

**Fig. (1):**
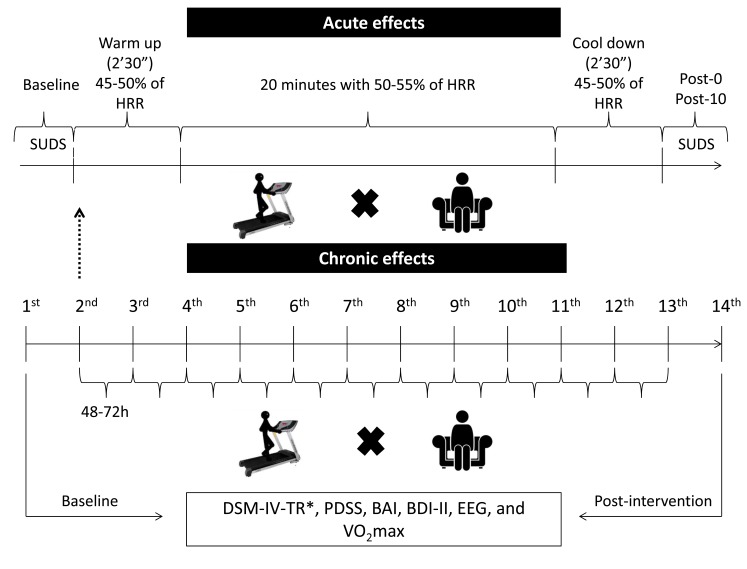
Schematic representation of the experimental procedures.

**Fig. (5):**
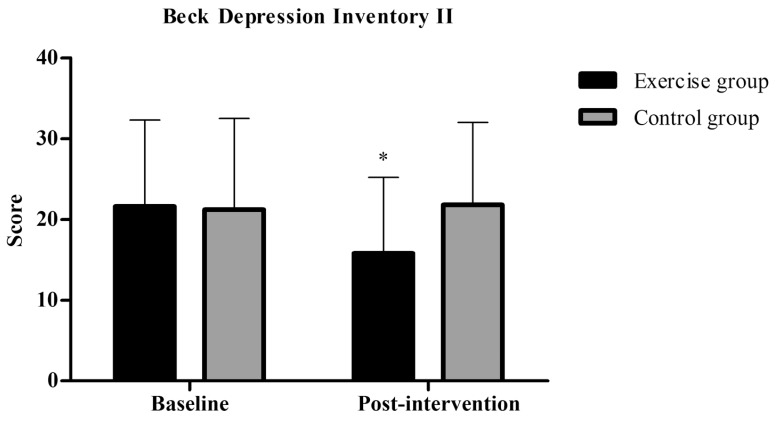
Between and within-group comparisons for BDI-II.

**Table 1:** Descriptive data at baseline.

Variables	Baseline		-
Exercise Group (n=5) (M±SD)	Control Group (n=5) (M±SD)	P
Age	36.4±3.5	42±8.4	0.20
Body weight (kg)	81±13.6	64±4.2	0.03*
Height (cm)	168.6±7.1	167.6±5.5	0.81
SUDS	2.4±1.1	2.2±0.8	0.76
PDSS	13.8±2.4	13.2±3.4	0.76
BAI	29.6±7.4	24.2±9.1	0.33
BDI-II	21.6±10.7	21.2±11.3	0.95
VO2max	35.4±2.7	32.2±2.2	0.08
Resting EEG asymmetry	-0.0295	-0.0130	0.75

Legend - Kg = kilogram; cm = centimeter; SUDS = Subjective Units of Distress Scale; PDSS = Panic Disorder Severity Scale; BAI = Beck Anxiety Inventory; BDI = Beck Depression Inventory-II; VO2max = maximum consumption oxygen in mL.kg-1.min-1; *Difference between groups at baseline.

**Table 2:** Between and within-group comparisons and effect size analysis for all outcome measures.

Variables	Exercise Group (EG)		Control Group (CG)		Effect Sizes		
Baseline (M±SD)	Post-intervention (M±SD)	Baseline	Post-intervention (M±SD)	Between-group	Within EG	Within CG
PDSS	13.8±2.4	13.0±2.0	13.2±3.4	13.0±3.3	0	0.75	0.18
BAI	29.6±7.4	24.0±7.1	24.2±9.1	24.2±8.2	0.02	1.40	0
BDI-II	21.6±10.7	15.8±9.4	21.2±11.3	21.8±10.2	-0.61	1.06	0.21
VO2max	±2.7	38.3±2.2	32.2±2.2	32.3±2.7	2.43	4.15	0.16
Resting EEG asymmetry	-0.0295±0.08	-0.0280±0.04	-0.0130±0.07	-0.0553±0.05	-0.60	0.02	0.51

Legend - PDSS = Panic Disorder Severity Scale; BAI = Beck Anxiety Inventory; BDI = Beck Depression Inventory-II; VO_2_max = maximum consumption oxygen in mL.kg^-1^.min^-1^.

## LIST OF ABBREVIATIONS

PD: = Panic Disorder
PAs: = Panic Attacks
CBT: = Cognitive Behavioural Therapy
CO_2_: = Carbon dioxide
CCK-4: = Cholecystokinin-tetrapeptide
P &A: = Panic and Agoraphobia Scale
VO_2_max: = Maximal oxygen consumption
EEG: = Electroencephalographic
BAI: = Beck Anxiety Inventory
BDI-II: = Beck Depression Inventory-II
PDSS: = Panic Disorder Severity Scale
SUDS: = Subjective Units of Distress Scale
μV: = MicroVolts
Hz: = Hertz
KΩ: = KOhms
FFT: = Fast Fourier Transform
ACSM: = American College of Sports Medicine
API: = Acute Panic Inventory
rpm: = Rotations per minute
HAM-A: = Hamilton Anxiety Scale
